# Resveratrol Mitigates Noise‐Induced Cochlear Damage and Delays Hearing Loss in Wistar Rats

**DOI:** 10.1155/bmri/7605759

**Published:** 2026-06-30

**Authors:** Ana Carolina Odorizzi Zica, Gabriela Guenther Ribeiro Novanta, Maria Luiza Queiroz Sampaio, Andressa Caram, Rafael Rocha de Andrade, Selma Aparecida Souza Kuckelhaus, Lucieny Silva Martins Serra, André Luiz Lopes Sampaio

**Affiliations:** ^1^ University of Brasilia, Brasilia, Brazil, unb.br; ^2^ Catholic University of Brasilia, Brasilia, Brazil, ucb.br

**Keywords:** hearing loss, noise, otoacoustic emissions, resveratrol, Wistar rats

## Abstract

**Introduction:**

This study was aimed at evaluating the potential otoprotective effects of resveratrol (Changsha Huir Biological‐Tech) on noise‐induced hearing loss (NIHL) in a Wistar rat model.

**Methods:**

The study employed a randomized controlled design. The noise used for stimulation was white noise, centered at a frequency of 4000 Hz (3564–4490 Hz), with an intensity of 100 dB SPL, for a period of 10 consecutive days at 8 h/day. Thirty rats were randomly assigned to three groups: a control nonexposed‐to‐noise group (*n* = 10) that received a vehicle solution of DMSO (dimethyl sulfoxide) (Sigma‐Aldrich), a noise‐exposed study group (*n* = 10) that received a vehicle solution of DMSO, and a resveratrol study group that was exposed to noise and received 10 mg/kg/day of resveratrol. All groups underwent distortion product otoacoustic emission (DPOAE) and auditory brainstem response (ABR) assessments at three time points: initial examination (D0), examination after 10 consecutive days of noise exposure (D15), and examination after 15 days of acoustic rest (D30). Histological evaluations were conducted on cochlear tissues after euthanasia. Statistical analyses were performed using Prism software to determine differences between groups and across time points.

**Results:**

The amplitude values, signal‐to‐noise ratios of DPOAE, ABR thresholds, and cochlear morphology between the experimental and control groups were significantly different from each other.

**Conclusion:**

The findings suggest that resveratrol exerts a partial otoprotective effect, possibly delaying the onset of NIHL. However, further research, particularly molecular research, is needed to better understand its therapeutic mechanism and its specific impact on auditory metabolic processes.

## 1. Introduction

Noise exposure constitutes one of the main public and environmental health problems in contemporary society, with implications that transcend the work environment. In the occupational context, noise is an omnipresent physical agent, responsible for noise‐induced hearing loss (NIHL), one of the most common and irreversible occupational diseases. However, the deleterious effects of noise are not limited to hearing; studies confirm its association with a range of nonauditory outcomes, including cardiovascular disorders such as hypertension and an increased risk of myocardial infarction, as well as psychophysiological effects such as chronic stress, sleep disturbances, anxiety, and impaired cognitive performance [1, 2]. This continuous exposure, both at work and in daily urban life, represents a significant allostatic load, negatively impacting individuals′ general health and quality of life.

In parallel with occupational and environmental exposure, the impact of recreational noise, especially associated with the use of personal audio devices such as headphones, is emerging with increasing concern. The World Health Organization (WHO) warns that over 1 billion young people and young adults are at risk of developing permanent hearing loss and tinnitus due to unsafe listening practices. The use of headphones for prolonged periods and at high volumes can expose the auditory system to sound pressure levels (SPLs) that frequently exceed safety limits (above 85 decibels [dB]). This voluntary and frequent exposure causes cumulative and irreversible hearing damage, anticipating the onset of auditory deficiencies that, in the past, were more common in elderly populations or those exposed to industrial noise, thus configuring a new and alarming paradigm of risk to global auditory health.

Evidence indicates that oxidative stress is a key element that may be involved in the pathogenesis of NIHL [3]. It is essentially characterized by an imbalance between the production of reactive oxygen species (ROS) and antioxidant defenses. In this sense, the use of antioxidants to reduce oxidative stress caused by ROS may be an effective step in the prevention of this disease [4]. Various antioxidant substances have been used in scientific research, such as vitamins A, C, and E, magnesium [5, 6], styrene [7], melatonin [8, 9], myricetin, and resveratrol [10–15].

Resveratrol, a polyphenol present in many plant foods, is widely known for its antioxidant and anti‐inflammatory properties and is believed to have therapeutic potential against many neurodegenerative diseases and metabolic disorders [14]. This substance has important biological activities, including inhibition of lipid peroxidation, copper chelation, free radical scavenging, alteration of eicosanoid synthesis, inhibition of platelet aggregation, modulation of lipid metabolism, anticancer activity, estrogenic activity, cardioprotection, and neuroprotection [11].

In research on the possible effects of resveratrol on the mitochondrial respiratory chain in rat brains, scientists discovered that it decreases the activity of Complex III by competing with coenzyme Q [15]. This property is particularly interesting because this complex is the site where ROS are generated. By decreasing the activity of Complex III, resveratrol not only opposes the production of ROS but also eliminates them. Furthermore, research has shown that resveratrol increases nicotinamide adenine dinucleotide (NAD) levels and is considered a potent agonist of Sirtuin 1 (SIRT1). SIRT1 is a highly conserved NAD‐dependent deacetylase protein, known for its protective effects against a wide range of neurological disorders [13, 16, 17]. Resveratrol also stimulates a key enzyme in the brain known as MAP kinase, which is involved in neural regeneration and protection from damage caused by systemic injection of the excitotoxin kainic acid [18].

This study hypothesized that resveratrol, administered preventively, attenuates auditory and structural damage induced by intense noise in Wistar rats, and its main objective was to evaluate the effect of resveratrol in preventing dysfunction of inner ear structures in Wistar rats exposed to high levels of sound pressure.

## 2. Materials and Methods

This was an experimental, prospective, controlled, and randomized study conducted at the Faculty of Medicine of the University of Brasilia. The sample consisted of 30 male Wistar rats (40 days old), and the study was approved by the Animal Use Ethics Committee (SEI No. 23106.001211/2020‐39) and was conducted in accordance with the guidelines of the National Institute of Health (NIH) and the National Council for the Control of Animal Experimentation (CONCEA).

### 2.1. Study Groups

• Resveratrol study group (*n* = 10): animals that were exposed to noise and received 10 mg/kg/day of resveratrol (Changsha Huir Biological‐Tech) previously diluted in DMSO (dimethyl sulfoxide) (Sigma‐Aldrich).
• Noise‐exposed study group (*n* = 10): animals that were exposed to noise and received a vehicle solution of DMSO.
• Control nonexposed‐to‐noise group (*n* = 10): animals that were not exposed to noise and received a vehicle solution of DMSO.

**Figure 1:**
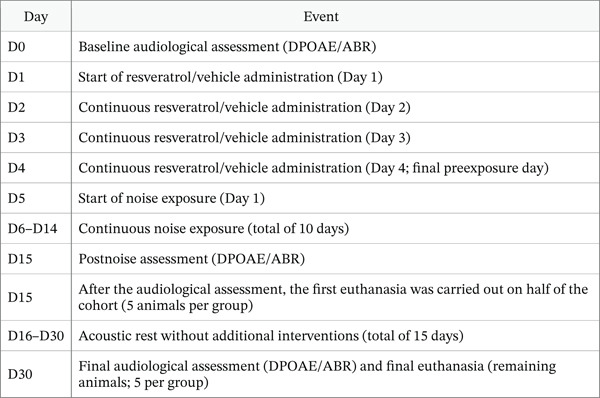
Experimental timeline summarizing all procedures, including baseline audiological assessments (D0), resveratrol/vehicle administration (D1–D4), noise exposure period (D5–D14), postnoise evaluation and partial euthanasia (D15), acoustic rest (D16–D30), and final assessments and euthanasia (D30).

Figure 1 summarizes the complete experimental timeline, providing a concise visual overview of all procedures, assessments, and euthanasia time points across the study.

### 2.2. Treatment

The animals received daily intraperitoneal applications of the solutions for a period of 14 days. The treatment began 4 days before the start of noise exposure (D1–D4), and the solutions were prepared immediately before each application. For the procedures, intraperitoneal anesthesia composed of ketamine hydrochloride (65 mg/kg [50 mg/mL]) and xylazine (6.5 mg/kg [20 mg/mL]) was administered. Animals that showed abnormal otoscopic findings or an absence of response (amplitude or negative signal‐to‐noise ratio [SNR]) in distortion product otoacoustic emission (DPOAE) at all analyzed frequencies, from 3000 to 12,000 Hz, were excluded from the study. Audiological evaluations (DPOAE and ABR [auditory brainstem response]) were conducted at three time points: initial examination (D0), after the end of noise exposure (D15), and after 15 days of acoustic rest (D30).

### 2.3. DPOAE Protocol

DPOAE examinations were performed using the OtoRead Evoked Otoacoustic Emissions device from Interacoustics. To obtain the distortion product (2F1‐F2), two pure tones were used at an F2/F1 ratio of 1.22, presented at an average intensity of 65 dB SPL (decibels sound pressure level) for F1 and 55 dB SPL for F2. The evaluation was performed bilaterally in all animals, in three measurements, alternating sides (right and left) at each evaluation. For data analysis, the average of the measurements performed was used. The primary metric used in the analysis of DPOAE was the amplitude of the 2F1‐F2 distortion product, expressed in dB SPL. Negative amplitudes may occur in DPOAE recordings and represent responses below the 0 dB SPL reference, which is physiologically acceptable. Although amplitude is the primary metric of interest, all measurements were first evaluated for their SNR, calculated for each frequency. An SNR ≥ 6 dB was used as the criterion for classifying a response as present, in accordance with international recommendations.

### 2.4. ABR Protocol

ABR was performed with the SmartEP equipment from Intelligent Hearing Systems, using subcutaneous needle electrodes positioned on the vertex (reference) and in the posterior region of both ears. Tone bursts (8 kHz) and rarefaction clicks were used as stimuli, presented at a rate of 21.1 per second, with up to 1000 averaged responses and an analysis window of 25 ms. Threshold search began at 80 dB nHL, with progressive intensity reduction (60, 40, 30, 20, 10, and 0 dB nHL) until wave disappearance. The electrophysiological auditory threshold was defined as the lowest intensity at which the II–III complex was identifiable. The SmartEP system (Intelligent Hearing Systems) uses precalibrated insert transducers whose intensity is expressed in dB nHL according to the device′s internal reference curve. This value represents a system‐specific normative threshold and is not equivalent to the nHL used clinically in humans. Before each experimental session, the equipment underwent routine verification, including checks of output levels and transducer integrity. The stimuli (click and tone burst 8 kHz) were delivered through insert earphones positioned in the animal′s external auditory canal, ensuring stable stimulus presentation. This protocol was applied only to the left ear. This decision was adopted to standardize the protocol and to avoid prolonged anesthesia time, which is particularly relevant in a model involving multiple manipulations. Previous studies have demonstrated strong interaural symmetry in ABR thresholds in rodents, reducing the likelihood of bias resulting from unilateral assessment [3–8, 10]. Moreover, DPOAE measurements were collected bilaterally and showed no between‐ear differences, further supporting the absence of significant functional asymmetry in the evaluated animals. The choice of the left ear was systematic and consistently applied across all experimental groups, thereby preventing differential bias.

### 2.5. Noise Exposure

The animals were exposed to noise inside an audiometric booth equipped with a system of eight loudspeakers, distributed on two levels, ensuring that each animal cage was directed toward a loudspeaker. The animals were exposed to white noise centered at 4 kHz (bandwidth 3564–4490 Hz) at an intensity of 100 dB SPL for 8 h/day. The noise was generated using the Noise Generator application and delivered inside an acoustic chamber through a system composed of a Pioneer MVH‐S218BT receiver, a Corzus HF‐404 amplifier module, and a CW‐12V80 power supply. Sound intensity was monitored using a DEC‐415 digital Sound Level Meter, previously calibrated with an acoustic calibrator before each experimental session. All measurements were conducted using A‐weighting (dB(A)) and the Slow time constant (1 s), in accordance with IEC 61672 standards for continuous noise measurement. To ensure exposure homogeneity, measurements were performed on each shelf and at four points inside each cage (one in each quadrant), always at approximately the height of the animals′ ears. Because the animals moved freely within the cages, this strategy minimized spatial variability and ensured that the sound field remained effectively uniform. The spectrum and frequency range of the generated noise were checked daily using the Noise Generator application to guarantee the stability of the acoustic stimulus throughout the entire protocol. This standardization ensured methodological reproducibility and minimized bias related to the animals′ position inside the cages.

### 2.6. Euthanasia

To reconcile functional assessments at multiple time points with histological analyses, the sample was divided into two euthanasia time points. Five animals per group were euthanized immediately after the postnoise audiological assessment (D15), whereas the remaining five animals were euthanized at the end of the acoustic rest period (D30).

### 2.7. Histological Processing

Animals were euthanized in two stages: immediately after noise exposure and after the acoustic rest period. Cardiac perfusion with 10% paraformaldehyde was performed for tissue fixation, and the heads were subsequently excised. Cochlear tissues were decalcified in a solution containing 5% nitric acid and 0.78% EDTA, processed, and embedded in Paraplast Plus for block preparation in sagittal and transverse planes. Histological sections (thickness of 6 *μ*m) were obtained by microtomy and stained with hematoxylin and eosin (H&E). For each ear, approximately 50 slides of serial sections were produced. All slides underwent a preliminary screening under light microscopy, and from this triage, we selected the three sections with the best morphological preservation and anatomical orientation for quantification. This approach ensured consistency across animals and reproducibility of the analysis. Cell counting was performed by a blinded examiner.

### 2.8. Histological Analysis

Images were acquired using the Aperio ScanScope system and analyzed with ImageScope software (Version 11.2.0.780) by a blinded evaluator at 20× and 40× magnification. The single‐blind photomicrographic analysis quantified the density of viable cells within predefined regions of interest: 80 *μ*m^2^ (spiral ganglion), 80 *μ*m^2^ (limbus), 50 *μ*m^2^ (spiral ligament), and 100 linear *μ*m (stria vascularis).

### 2.9. Statistical Analysis

The sample size was defined by CEUA/UnB. Although the study collected outcomes across multiple time points (D0, D15, and D30) and frequencies (3–12 kHz), the experimental structure prevented the use of statistical approaches appropriate for repeated‐measures data, such as RM‐ANOVA or linear mixed‐effects models. Because half of the animals were intentionally euthanized immediately after noise exposure (D15) to obtain histological material and the remaining half only at the end of the study (D30), individual subjects did not contribute complete observations across all time points, resulting in an unbalanced and incomplete longitudinal matrix. Consequently, analyses were performed using between‐group and between‐time comparisons. For DPOAE, ABR, and histological endpoints, the choice between parametric and nonparametric procedures was made according to the nature of each comparison; parametric comparisons were analyzed using one‐way ANOVA followed by Newman–Keuls or Holm–Sidak post hoc tests, whereas nonparametric comparisons were analyzed using Kruskal–Wallis tests followed by Dunn′s test with Bonferroni correction. DPOAE measurements were first compared between the right and left ears; because no significant differences were found, values were pooled, and the animal was considered the statistical unit to avoid pseudoreplication. Effect sizes were calculated using Cohen′s *d* with Hedges′ *g* bias correction, and 95% confidence intervals were computed to complement the *p* values and quantify the magnitude and precision of group differences. Differences were considered statistically significant at *p* < 0.05. All analyses were performed using Prism Version 5 (GraphPad Software, San Diego, California, United States).

## 3. Results

### 3.1. DPOAE

A paired analysis was performed between the right and left ears at D0 and D30. Given the uniformity found (Mann–Whitney test,*p* = 0.116[D0],*p* = 0.260[D30]), the ears were grouped. No differences were observed between median amplitudes (*p* > 0.05 at all analyzed frequencies) in the comparison between D0, D15, and D30 (*p* = 0.087), confirming that the control nonexposed‐to‐noise group remained stable throughout the study.

**Figure 2:**
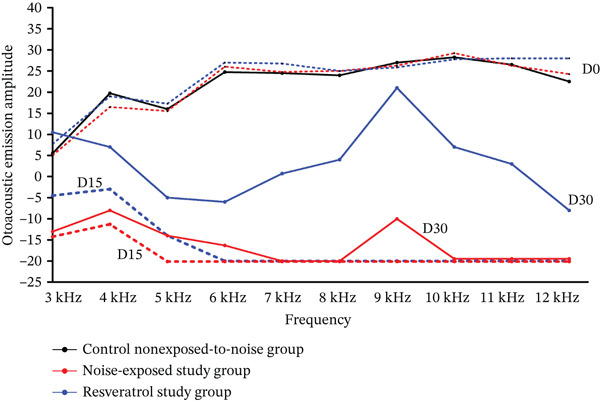
Frequency‐specific variation in DPOAE amplitudes across D0, D15, and D30 for each experimental group.

• D0 analysis (initial examination): The comparison revealed no significant differences between the control nonexposed‐to‐noise group, noise‐exposed study group, and resveratrol study group (*p* = 0.083) in the median amplitude across the 3–12‐kHz frequency range, as shown in Table 1.
• D15 analysis (after noise exposure): In the postexposure analysis, when comparing the control group with the noise‐exposed and resveratrol study groups, the analysis revealed a reduction in DPOAE amplitudes (*p* < 0.001) across the 5–12‐kHz frequency range, with no significant differences at 3 and 4 kHz (*p* = 0.688 and *p* = 0.548). Furthermore, the comparison indicated that this reduction was homogeneous between the two experimental groups (noise‐exposed and resveratrol, *p* = 0.716), as shown in Table 1.
• D30 analysis (after acoustic rest): In the postrest analysis, when comparing the control group with the noise‐exposed and resveratrol study groups, the analysis showed that differences were maintained across the 6–12‐kHz frequency range (*p* < 0.001), with no significant differences at 3, 4, or 5 kHz (*p* = 0.397, *p* = 0.274, and *p* = 0.198). However, the comparison revealed greater recovery in the resveratrol study group at 7–12 kHz (*p* < 0.001), as shown in Table 1 and Figure 2.

**Table 1:** Median amplitude variation (standard deviation [SD]) of DPOAE in comparison between groups: Control nonexposed‐to‐noise group (CN), noise‐exposed study group (NO), and resveratrol study group (RE) at D0, D15, and D30 time points.

Freq. (kHz)	Time (days)	Median/SD Amplitude (median)			Statistical analysis ($\begin{array}{c} p=0.083 \end{array}$)	Time (days)	Median/SD Amplitude (median)			Statistical analysis ($\begin{array}{c} p<0.001 \end{array}$)	Time (days)	Median/SD Amplitude (median)			Statistical analysis ($\begin{array}{c} p<0.001 \end{array}$)
		Control nonexposed‐to‐noise group (CN)	Noise‐exposed study group (NO)	Resveratrol study group (RE)			Control nonexposed‐to‐noise group (CN)	Noise‐exposed study group (NO)	Resveratrol study group (RE)			Control nonexposed‐to‐noise group (CN)	Noise‐exposed study group (NO)	Resveratrol study group (RE)	
3	D0	5.12 ± 5.40	4.19 ± 4.61	7.536 ± 5.84	CN ≈ NO ≈ RE	D15	9.00 ± 5.14	−12.24 ± 7.36	−1.91 ± 5.27	CN ≈ NO ≈ RE	D30	10.19 ± 6.33	−9.43 ± 9.70	8.92 ± 5.41	CN ≈ NO ≈ RE
		5.50	5.00	7.75			9.50	−14.25	−4.50			12.25	−13.00	10.50	
4		18.63 ± 5.59	15.83 ± 3.32	17.09 ± 4.85	CN ≈ NO ≈ RE		20.81 ± 5.49	−10.37 ± 3.79	−10.37 ± 3.79	CN ≈ NO ≈ RE		20.56 ± 6.98	−7.30 ± 7.91	7.00 ± 10.44	CN ≈ NO ≈ RE
		19.75	16.50	19.00			22.15	−11.30	−3.00			22.75	−8.00	7.00	
5		16.00 ± 3.66	15.13 ± 4.91	16.27 ± 4.63	CN ≈ NO ≈ RE		19.04 ± 5.29	−18.17 ± 2.90	−13.86 ± 5.60	CN > NO/RE ^∗^		17.60 ± 7.04	−13.56 ± 4.75	−1.85 ± 10.24	CN ≈ NO ≈ RE
		16.00	15.50	17.25			19.90	−20.00	−14.00			20.15	−14.00	−5.00	
6		24.25 ± 3.88	25.06 ± 3.09	26.58 ± 4.10	CN ≈ NO ≈ RE		24.38 ± 3.82	−19.34 ± 1.20	−17.25 ± 5.31	CN > NO/RE ^∗^		24.38 ± 3.82	−15.15 ± 6.08	−0.50 ± 11.50	CN > RE/NO ^∗^
		24.75	26.00	27.00			24.75	−20.00	−20.00			25.00	−16.30	−6.00	
7		24.06 ± 3.35	24.67 ± 2.74	26.44 ± 3.75	CN ≈ NO ≈ RE		24.94 ± 4.20	−19.98 ± 0.10	−17.25 ± 5.31	CN > NO/RE ^∗^		23.13 ± 6.08	−15.47 ± 8.28	2.52 ± 10.80	CN > RE ^∗^ > NO ^∗^
		24.50	24.75	26.75			24.75	−20.00	−20.00			23.75	−20.00	0.70	
8		23.94 ± 2.51	24.71 ± 1.79	25.58 ± 2.99	CN ≈ NO ≈ RE		24.13 ± 3.61	−19.52 ± 1.50	−17.21 ± 5.18	CN > NO/RE ^∗^		23.63 ± 4.00	−15.02 ± 9.98	2.18 ± 12.22	CN > RE ^∗^ > NO ^∗^
		24.00	25.00	25.00			23.75	−20.00	−20.00			24.25	−20.00	4.00	
9		26.94 ± 2.00	25.58 ± 2.88	26.65 ± 3.23	CN ≈ NO ≈ RE		26.88 ± 4.38	−19.32 ± 1.99	−17.71 ± 4.22	CN > NO/RE ^∗^		26.81 ± 4.40	−13.40 ± 11.92	0.42 ± 17.75	CN > RE ^∗^ > NO ^∗^
		27.00	26.25	25.85			27.00	−20.00	−20.00			28.00	−20.00	1.50	
10		27.44 ± 4.53	29.38 ± 1.68	27.88 ± 3.61	CN ≈ NO ≈ RE		26.13 ± 4.97	−19.48 ± 1.27	−17.61 ± 4.82	CN > NO/RE ^∗^		25.81 ± 4.52	−14.33 ± 9.74	3.85 ± 16.67	CN > RE ^∗^ > NO ^∗^
		28.25	29.25	27.75			26.00	−20.00	−20.00			27.00	−19.50	7.00	
11		26.34 ± 4.98	27.48 ± 2.76	27.10 ± 4.43	CN ≈ NO ≈ RE		22.63 ± 6.28	−19.63 ± 0.858	−17.79 ± 5.25	CN > NO/RE ^∗^		24.50 ± 3.57	−14.69 ± 10.77	−1.54 ± 19.19	CN > RE ^∗^ > NO ^∗^
		26.50	26.25	28.00			22.00	−20.00	−20.00			26.00	−19.50	3.00	
12		23.88 ± 7.51	25.46 ± 3.29	26.54 ± 5.97	CN ≈ NO ≈ RE		22.98 ± 5.27	−19.48 ± 1.49	−18.6 ± 3.21	CN > NO/RE ^∗^		22.38 ± 3.14	−14.69 ± 10.77	−1.54 ± 19.19	CN > RE ^∗^ > NO ^∗^
		22.50	24.25	28.00			24.00	−20.00	−20.00			22.75	−19.50	−8.00	

^∗^Statistical significance determined for p < 0.05.

Figure 2 demonstrates the variability in DPOAE signal amplitude results across the control, resveratrol study, and noise‐exposed study groups. It is possible to verify that in the initial examination, the three research groups were homogeneous. After the end of noise exposure, both the noise‐exposed study group and the resveratrol study group showed a reduction in DPOAE signal amplitude thresholds. It is also observed that after the acoustic rest period, both study groups showed threshold recovery, but the resveratrol study group showed visibly greater recovery.

### 3.2. Effect Size Analysis Across Time Points

Effect size analysis using Cohen′s *d* with Hedges′ *g* bias correction and 95% confidence intervals showed a consistent and biologically meaningful pattern across the study period. At D0, effect sizes were small (approximately −0.9 to 0.7), and confidence intervals crossed zero, indicating no relevant preexisting differences among groups. At D15, effect sizes became very large (approximately −5.1 to 9.7), with confidence intervals excluding zero across most frequencies, confirming a strong noise‐induced functional impairment. In contrast, the resveratrol study group showed smaller effects relative to the noise‐only group, supporting a protective effect across the cochlear frequency range. By D30, effect sizes remained substantial (approximately −2.0 to 3.8), with persistent large contrasts involving the noise‐only group and smaller‐to‐moderate effects in comparisons involving the resveratrol study group, suggesting partial recovery and sustained otoprotection.

### 3.3. ABR

In the analysis of the results obtained by ABR with click and tone burst (8 kHz) stimuli, the control nonexposed‐to‐noise group showed stable ABR thresholds (click: *p* = 1.000; tone burst 8 kHz: *p* = 0.8736) when compared with examinations at different time points (D0, D15, and D30).

• D0 analysis (initial examination): In the initial analysis, the comparison revealed no significant differences among the control, noise‐exposed study, and resveratrol study groups for either the click stimulus (*p* = 1.000) or the tone burst (8 kHz) stimulus (*p* = 0.873), indicating baseline homogeneity among the groups, as shown in Table 2.
• D15 analysis (after noise exposure): In the postexposure analysis, the comparison of the control (nonexposed‐to‐noise) group with the noise‐exposed and resveratrol study groups showed significant differences (*p* < 0.001) in both stimuli (click and tone burst 8 kHz), with increased thresholds following noise exposure. Moreover, the analysis revealed no significant difference in the median amplitude between the two experimental groups (noise‐exposed and resveratrol), indicating that the threshold increase was homogeneous across both stimuli (click: *p* = 0.784; tone burst: *p* = 0.560), as shown in Table 2.
• D30 analysis (after acoustic rest): In the postrest analysis, the comparison of the control group with the study groups showed that, in the noise‐exposed study group, significant differences (*p* < 0.001) were maintained for both click and tone burst (8 kHz) stimuli. In contrast, the resveratrol study group exhibited threshold recovery, with a significant difference remaining only for the tone burst stimulus (*p* < 0.001; click: *p* = 0.098), as shown in Table 2.

**Table 2:** Median variation of ABR in click and tone burst (8 kHz) stimuli by group at D0, D15, and D30 time points.

ABR	Time (days)	Median/SD Auditory threshold (median)			Statistical analysis (click: $\begin{array}{c} p=1.000 \end{array}$; TB: $\begin{array}{c} p=0.087 \end{array}$)	Time (days)	Median/SD Auditory threshold (median)			Statistical analysis ($\begin{array}{c} p<0.001 \end{array}$) ^∗^	Time (days)	Median/SD Auditory threshold (median)			Statistical analysis ($\begin{array}{c} p<0.001 \end{array}$) ^∗^
		Control nonexposed‐to‐noise group (CN)	Noise‐exposed study group (NO)	Resveratrol study group (RE)			Control nonexposed‐to‐noise group (CN)	Noise‐exposed study group (NO)	Resveratrol study group (RE)			Control nonexposed‐to‐noise group (CN)	Noise‐exposed study group (NO)	Resveratrol study group (RE)	
Click	D0	5 ± 5.47	7.69 ± 5.99	4.44 ± 5.2	CN ≈ NO ≈ RE	D15	5 ± 5.47	29.23 ± 12.56	18.89 ± 9.28	CN < NO/RE ^∗^	D30	5 ± 5.47	21.0 ± 7.37	10.0 ± 5.77	CN < NO ^∗^ > RE
		05.00	10.00	00.00			05.00	20.00	20.00			05.00	20.00	10.00	
TB (8 kHz)		15 ± 5.47	22.31 ± 8.32	18.89 ± 6.0	CN ≈ NO ≈ RE		16.67 ± 8.16	53.85 ± 14.46	54.44 ± 15.9	CN < NO/RE ^∗^		16.67 ± 5.16	46.0 ± 15.06	38.0 ± 8.36	CN < NO/RE ^∗^
		15.00	20.00	20.00			15.00	50.00	60.00			20.00	40.00	40.00	

^∗^Statistical significance determined for p < 0.05.

**Figure 3:**
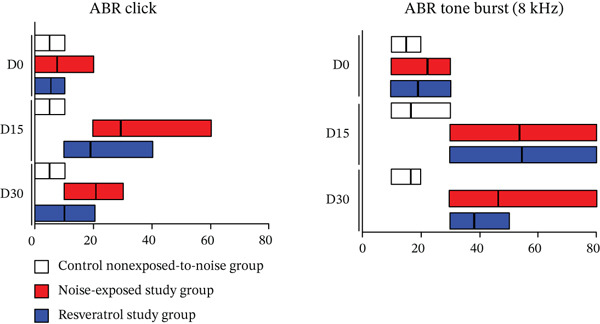
Boxplots of the variation of ABR click and tone burst (8 kHz) test thresholds obtained in the evaluations of the initial examination (D0), examination after noise exposure (D15), and examination after acoustic rest (D30) in the control nonexposed‐to‐noise, noise‐exposed study, and resveratrol study groups.

Figure 3 illustrates the variability in median ABR thresholds with click and tone burst (8 kHz) stimuli among the control, resveratrol study, and noise‐exposed study groups. Homogeneity among the three groups was observed in the initial examination. Following the noise exposure period, both the noise‐exposed study group and the resveratrol study group showed increased thresholds for both types of stimuli. After the acoustic rest period, both study groups showed threshold recovery for click and tone burst (8 kHz) stimuli, with the resveratrol study group showing visibly greater recovery.

### 3.4. Structural Evaluation of the Cochlea

**Figure 4:**
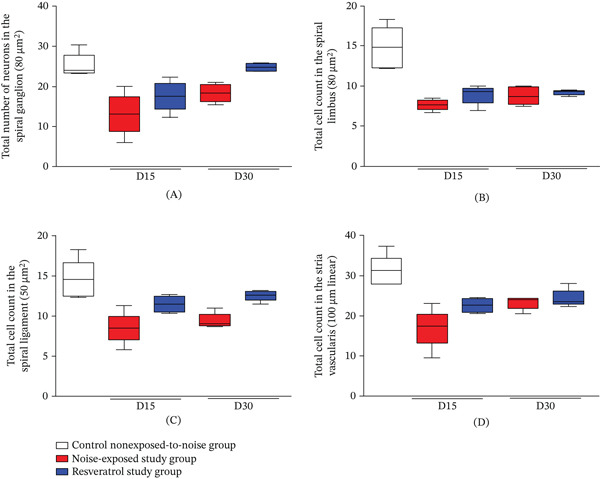
Total cells quantified per area in cochlear tissues: (A) spiral ganglion (80 *μ*m^2^), (B) limbus (80 *μ*m^2^), (C) spiral ligament (50 *μ*m^2^), and (D) stria vascularis (100 linear *μ*m).

Because semiquantitative analysis of outer and inner hair cells was not feasible, structural analysis was performed on cochlear tissues (spiral ganglion, limbus, spiral ligament, and stria vascularis). The results showed higher cell counts in the spiral ganglion, limbus, stria vascularis, and spiral ligament of animals in the control group compared with the noise‐exposed study group (*p* < 0.001) (Figure 4). The resveratrol study group partially reversed the noise‐induced cell damage in the limbus (*p* < 0.001), spiral ligament (*p* = 0.03), and stria vascularis (*p* < 0.001), as shown in Figure 4.

**Figure 5:**
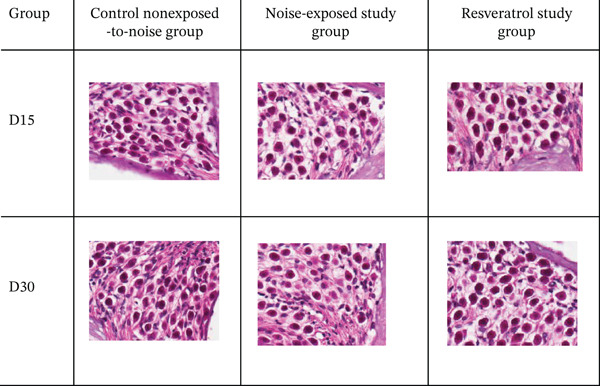
Representative photomicrographs of the results of cell area quantification in the cochlear tissues of the spiral ganglion. Staining: hematoxylin and eosin.

Specifically in the spiral ganglion, resveratrol treatment reversed the reduction in ganglion neuron density (neurons per 80 *μ*m^2^) observed in the noise‐exposed study group and restored values to levels comparable to those in the control group (*p* = 0.2302). These results are illustrated in Figure 5, which shows the variability in the total number of ganglion neurons after the noise exposure and acoustic rest periods.

## 4. Discussion

### 4.1. Context of NIHL and Potential of Antioxidants

Current strategies for NIHL prevention include the use of personal protective equipment and environmental noise control interventions; however, despite being restrictive and widely enforced, they are not sufficient. We are facing a growing number of people with hearing loss caused by constant noise exposure, which makes the search for therapies capable of preventing the negative impact of noise on the auditory system even more important [19]. Antioxidants have emerged as a promising approach, as previous studies suggest that they may counteract the excessive production of free radicals in the noise‐exposed cochlea and/or noise‐induced cochlear vasoconstriction, thereby mitigating the consequences of NIHL [4, 5, 7–9, 11, 14, 20, 21].

Several studies have been conducted using Wistar rats, as this animal model has proven effective in auditory system research due to its structural similarities with the human ear [5, 6, 8–11, 21–24].

### 4.2. Rationale for Resveratrol Use and Methodological Details of the Study

A study published in 2020 described that resveratrol exhibits a series of therapeutic benefits, including anti‐inflammatory, antioxidant, antiplatelet, antihyperlipidemic, anticancer, cardioprotective, immunomodulatory, vasorelaxant, and neuroprotective effects. This polyphenol appears to alleviate the main risk factors for cardiovascular diseases, as it can improve endothelial function, eliminate ROS, reduce inflammation, inhibit platelet aggregation, and improve the lipid profile and other key factors that can promote atherosclerosis. Furthermore, mechanisms associated with oxidoreduction have been implicated as potential pathways by which resveratrol exerts its protective effects. These oxidoreduction‐associated mechanisms include the preservation of mitochondrial function under hypoxia/reoxygenation‐induced oxidative stress, upregulation of antioxidant enzymes such as peroxidase and superoxide dismutase, and modulation of nitric oxide production [25]. Studying the otoprotective effect of resveratrol may indicate a path in the search for pharmacological therapies capable of neutralizing and/or delaying the effects of NIHL. In this study, the antioxidant was administered for 4 days before the onset of noise exposure and continued throughout the entire 10‐day exposure period. This administration protocol was designed to allow the antioxidant and anti‐inflammatory properties of resveratrol to act preventively.

A worker is typically exposed to noise for an average of 8 h daily; thus, in this study, the animals were exposed for this duration over a 10‐day period to reproduce a condition similar to a worker′s daily exposure, and this approach is supported by literature highlighting that short exposures are convenient for creating inner ear damage in basic morphology and function studies but provide limited insight into the mechanisms of NIHL in humans. The authors also note that the stabilization of hearing loss in experimental research occurs around the fifth day of exposure [26].

The choice of 100‐dB intensity was based on a previous study [24] and is consistent with reports indicating that noise levels between 100 and 110 dB are required to induce a stable, measurable hearing loss in rat models [27]. The choice of the noise frequency range, centered at 4 kHz, was guided by studies showing that hearing loss typically occurs one octave above the exposure frequency, thereby optimizing outcome detection within the technical limits of the equipment used in this study [28].

### 4.3. Analysis of Functional Results: DPOAE and ABR

DPOAE amplitude and SNR results confirmed that the intended NIHL model was successfully established, as the noise‐exposed study group showed reduced DPOAE amplitudes relative to the control nonexposed‐to‐noise group, particularly at frequencies from 5 to 12 kHz following noise exposure, which persisted at 6–12 kHz after the acoustic rest period, consistent with previous reports [10, 29–37]. In our study, the configuration of hearing loss could not be directly related to the frequency of the noise used. These findings suggest that exposure duration may also play a critical role in determining the tonotopic pattern of cochlear damage, underscoring the importance of exposure parameters in NIHL modeling.

Regarding the results found in DPOAE, although the alterations remained significant after the acoustic rest period, the results of the resveratrol study group showed visible recovery of thresholds when compared with the noise‐exposed study group. This finding was further supported by a significant difference in the signal amplitude between the groups at frequencies from 7 to 12 kHz after the acoustic rest period.

Beyond statistical significance, the effect size pattern supports the biological relevance of the findings. The small baseline effects confirm group comparability, whereas the very large effects at D15 indicate substantial noise‐induced cochlear dysfunction. The consistently smaller effects in the resveratrol study group suggest that the intervention reduced the magnitude of injury rather than merely altering statistical significance. Together, these results indicate sustained otoprotection and partial functional recovery over time.

Analysis of ABR results showed that both the noise‐exposed and resveratrol study groups differed significantly from the control group for both click and tone burst (8 kHz) stimuli after the noise exposure and acoustic rest periods. However, the resveratrol study group exhibited a more pronounced recovery.

### 4.4. Analysis of Cochlear Histological Results

Regarding the histological analysis, all structures, namely, stria vascularis, spiral ligament, limbus, and spiral ganglion, showed differences compared with the control group after noise exposure and after the acoustic rest period; however, recovery was even more evident in the neuronal cells of the spiral ganglion, which became comparable to the total number of cells in the control group after acoustic rest. A study investigated the effects of resveratrol on Sprague‐Dawley rats that received daily kainate (KA) (8 mg/kg) administration for 5 days. Compared with the KA‐only group, coadministration of resveratrol (30 mg/kg/day) significantly attenuated neuronal damage, inhibited astrocyte and microglial activation, increased neuronal viability, and delayed apoptosis [38].

### 4.5. Protective Mechanisms and Limitations of Otoprotection

These results suggest that although resveratrol is a promising antioxidant, it does not completely neutralize the occurrence of NIHL; however, it may delay the onset of this condition. This temporary threshold shift was also reported in a previous study that used noise exposure as an ototoxic stimulus and resveratrol as an otoprotective agent. In the study conducted by Xiong et al., mice were exposed for 1 h to 120‐dB noise centered at 10 kHz. ABR analysis, performed immediately after exposure, concluded that there was no difference in responses between the groups exposed to noise, regardless of resveratrol use. However, in the 15 days after exposure analysis, improved recovery occurred at frequencies of 4 and 16 kHz in animals that used resveratrol. According to the authors, resveratrol, described as a SIRT1 activator, protected hearing from noise‐induced damage by increasing cochlear SIRT1 activity and consequently reducing oxidative stress.

Other studies have reported that, following noise exposure, oxidative stress readily affects auditory hair cells, stria vascularis cells, and spiral ganglion neurons, as these structures rely heavily on oxidative metabolism to meet the exceptionally high energy demands of mechanoelectrical transduction. This is consistent with the findings of the present study. These studies indicate that an excess of free radicals involves dysregulated redox reactions, damaging essential membrane lipids and proteins for auditory signal transduction, ultimately leading to cell death and NIHL [5–7, 34, 39, 40].

Although preclinical studies suggest that resveratrol may act through multiple antioxidant and mitochondrial pathways, the present investigation did not include the quantification of molecular markers, such as SIRT1, NOX3, and HSP70, lipid peroxidation products, or apoptotic proteins. In the absence of these biochemical measurements, it is not possible to determine whether the functional and histological improvements observed in the resveratrol study group were directly mediated by specific oxidative, inflammatory, or mitochondrial mechanisms. Despite these limitations, the combination of functional (DPOAE and ABR) and structural (histology) assessments provides robust evidence of partial protection by resveratrol in this model of noise‐induced cochlear injury. Future studies incorporating targeted molecular analyses will be essential to confirm or refute these mechanistic hypotheses.

### 4.6. Implications, Limitations, and Future Perspectives

The findings of this study suggest that the preventive administration of resveratrol offers a promising approach to mitigating the deleterious effects of noise on hearing, preventing oxidative stress–induced auditory degeneration. This evidence reinforces the potential of antioxidants as otoprotective agents, offering a path for the development of therapies capable of delaying or attenuating NIHL.

However, it is important to acknowledge the inherent limitations of this study. First, the lack of detailed molecular analysis limits a deeper understanding of the specific mechanisms through which resveratrol exerts its protective effect on cochlear structures. In addition, the relatively small number of animals per group may limit the generalizability of the results and the detection of more subtle effects. The single intraperitoneal dose of 10 mg/kg/day was selected based on consistent preclinical evidence demonstrating otoprotective effects of resveratrol within this pharmacological range, particularly in models of oxidative stress and cochlear injury induced by continuous noise exposure. This dose has been widely used in experimental studies due to its favorable profile of bioavailability, safety, and ability to reach sufficient tissue and mitochondrial concentrations to modulate antioxidant and inflammatory pathways. However, we acknowledge that the absence of a dose–response assessment limits the pharmacological extrapolation of our findings, preventing determination of whether lower or higher doses could produce superior, equivalent, or even dose‐dependent effects. Additionally, our protocol evaluated exclusively a preventive regimen administered during the noise exposure period, making it impossible to infer the therapeutic potential of resveratrol when administered after acoustic exposure.

Given these considerations, future research is crucial to further advance knowledge in this area. Studies incorporating gene expression analyses of key markers involved in oxidative stress and the sirtuin pathway, such as SIRT1, NOX3, and HSP70, are recommended to clarify the molecular mechanisms underlying resveratrol′s otoprotective effects. Investigating different dosing regimens and administration routes for resveratrol, as well as varying noise exposure durations, would also be valuable for optimizing its efficacy. Moreover, exploring the combined use of resveratrol with other otoprotective agents and translating these findings into larger preclinical models and ultimately clinical trials may pave the way for new therapeutic strategies to prevent NIHL in humans, with substantial potential to improve the quality of life of individuals exposed to intense noise.

## 5. Conclusion

The findings of this study indicate that resveratrol exerted a partial otoprotective effect in Wistar rats exposed to intense noise, as demonstrated by improved functional outcomes (DPOAE and ABR) and enhanced histological recovery compared with the noise‐exposed control group. Although it did not fully prevent NIHL, resveratrol may slow the progression of cochlear damage. Further studies, including molecular analyses, are needed to clarify its mechanisms of action and assess its translational potential.

## Author Contributions

**Ana Carolina Odorizzi Zica:** conceptualization, investigation, formal analysis, data curation, writing – original draft, writing – review and editing. **Gabriela Guenther Ribeiro Novanta:** conceptualization, investigation, formal analysis, data curation. **Maria Luiza Queiroz Sampaio:** investigation, writing – original draft. **Andressa Caram:** investigation. **Rafael Rocha de Andrade:** methodology, investigation. **Selma Aparecida Souza Kuckelhaus:** conceptualization, methodology, formal analysis. **Lucieny Silva Martins Serra:** conceptualization, formal analysis, supervision. **André Luiz Lopes Sampaio:** conceptualization, methodology, writing – review and editing, supervision, project administration.

## Funding

This research received financial aid from a scholarship provided by Fundação de Apoio à Pesquisa do Distrito Federal under Edict DPG No. 0011/2022. This study was financed in part by the Conselho Nacional de Desenvolvimento Científico e Tecnológico (CNPq) – Brazil through a doctoral fellowship (Process No. 141911/2025‐5).

## Conflicts of Interest

The authors declare no conflicts of interest.

## Data Availability

The data that support the findings of this study are available on request from the corresponding author. The data are not publicly available due to privacy or ethical restrictions.
